# Book Reviews

**Published:** 1824-03

**Authors:** 


### CRITICAL ANALYSIS

OF

### ENGLISH AND FOREIGN LITERATURE

RELATIVE TO THE VARIOUS BRANCHES OF

### Medical Science.

Quæ laudanda forent, et quæ culpanda, vicissim

Illa, prius, cretâ; mox hæc, carbone, *notamus*.—PERSIUS.

### DIVISION I.

### ENGLISH.

ART. I.—*Lectures on the General Structure of the Human Body, and on the Anatomy and Functions of the Skin; delivered before the Royal College of Surgeons in London, in the Courses for* 1823. By THOMAS CHEVALIER, F.R.S. F.S.A. F.L.S. & F.H.S. Surgeon Extraordinary to the King, and Professor of Anatomy and Surgery to the College.—8vo. pp. 277; seven Plates. London: Longman and Co. 1823.

WE will not flatter Mr. CHEVALIER by saying that this work is likely to add much to his already well-earned reputation; and yet it is prettily—in many parts even elegantly written, the style easy and flowing, the allusions occasionally happy, and the quotations apt: nevertheless, the three first Lectures especially, do not quite come up to the *beau ideal* we had formed in our own minds of what such discourses ought to be, considering the class of persons before whom they are delivered, and the chair from which they are read. In the first of these lectures, for example, many deep and important questions are propounded, each of them capable of being extended into a tolerable dissertation, the majority of which are however passed by, either unnoticed or touched upon in a very slight manner. Nearly twenty pages of this lecture are taken up in complimenting either the past or the present professors of the College, and the remainder is a sort of desultory description of the complex machinery of the human frame, a few sentences on the distinction between organization and life, with perhaps too frequent and earnest allusions to matters of a higher and more serious cast; the importance of which, although we are far from denying, or wishing to extenuate, yet we cannot see the necessity for insisting upon in works and discourses purely professional. But, as it is neither our wish nor intention to dwell longer than necessary upon what we must regard as somewhat below the standard of our author's former labours, we pass on to the consideration of that portion of the volume more especially devoted to a description of the common integument, which contains many features of novelty, the result of actual observation, of patient and laborious research, and great labour in the execution of the mechanical part.

We do not consider ourselves entitled to interrupt this narrative with any remarks of our own, considering it to be no more than just to give as clear a view of our author's opinions as our limits will permit, and leaving it to future observers to verify or refute the statements he has made: it is, however, to be remembered that the description which Mr. Chevalier has given of the component parts of the skin, is all derived from preparations made by him, with the assistance of his son; that imagination has no share in the account; and that a series of their preparations is deposited in the College museum, for the examination of the curious inquirer. It may, perhaps, be fairly questioned whether the diseases of the skin are likely to be better illustrated, or more certainly cured, in consequence of our author's researches; but, even should this not be the case, Mr. Chevalier has nothing to reproach himself; if he has succeeded in clearing up any of the doubtful points, or elucidating any of the obscurities of the anatomy of this important organ, he has performed his part; and the practical application of his discoveries, though not at present obvious, may nevertheless become available at some future period: as far as he has extended the boundaries of our knowledge, he is deserving of our gratitude.

Our author commences this subject by informing his auditors that he was for some time in doubt as to the subject of the remainder of his anatomical lectures, but at length it occurred to him that, as in the surgical part of the Course he intended to treat of those diseases which affect all structures, it would be a proper preliminary to consider, first of all, the simple structures themselves, and he determined to begin with the skin. He obviates the objection that might be made to considering so complicated a portion of the body as a simple structure, by observing that, "however complicated it may be, it is only so from an intertexture, or co-adaptation, of distinguishable substances; and, as far as we can separate them, we must be profited by the careful analysis that resolves them into the simple materials, which are thus blended or fitted together." (p. 99.)

Passing over two or three pages of matter irrelevant to the subject under review, we come to a description of the *cuticle*, or *epidermis*, and which does not differ from that usually given in all anatomical works: neither do we derive any new information respecting its constituent parts. We are told that Mr. Hatchett found it to consist chiefly of gelatine; and, as Sir H. Davy found silex to form a part of the cuticle of vegetables,

[quere, of *some* vegetables only?] our author seems inclined to think it might be worth while to inquire whether it does not form a part of animals also. The use of this we do not exactly perceive: it may, perhaps, gratify curiosity; it can scarcely be convertible to any useful purpose.

Our author next remarks upon the unprutrescible quality and strong preservative powers of this substance: a fact with which the ancient Egyptians were so well acquainted, as always to take it off from the body, in order that the bituminous substance might be imbibed; and that in all those parts where the cuticle has not been removed, and the bituminous impregnation has not taken place, the parts underneath appear to owe their preservation to the communication of the preserving power of the substance entering into those which adjoin, or are attached to them. All the functions of the skin are performed through its intervention; the bulbs, the hairs, the sebaceous glands, and the nails, are all kept by it in their proper situations. It is in a state of perpetual growth and decay, the exterior surface falling off in scabs or patches. It clothes innumerable papillæ and millions of transpiring and absorbing vessels; and, without a proper knowledge of it, neither the anatomy, physiology, nor pathology, of the cutis itself can be understood. The cuticle is nourished by vessels ramifying on the cutis, and appropriated to this purpose; and from these are derived the serous fluid, by which it is occasionally distended, even sometimes suddenly, as in scalds. It is always ultimately destroyed in consequence of this separation.

A few words are said as to the vitality of this membrane, which has been denied: our author is of opinion that it possesses vitality, but in a feeble degree. It is surely a trifling discussion; at all events, it is insensible. With the aid of the magnifying glass, we may detect much variety on its outer surface; for example, in the cheeks, nose, scrotum, palms of the hands, &c.: varieties, says our author, which are original, and not adventitious; the original texture being intended, however, to make provision for the occasional production and security of the adventitious, when and so long as it may be needed.

We now come to particulars.

" On the ends of the fingers and toes, and on the ball of the thumb, and on the under part of the heels, it presents a curvilinear appearance; whereas, in other parts, it is arranged in rugæ in various directions,—longitudinal, transverse, and angular. In the former, where the nails are inserted into it, it is evidently intended to preserve that degree of tenseness in the subjacent skin which is essential to the nicest exactitude and delicacy of the organ of touch; being stretched over the cutis, from one side of the nail to the other, somewhat as the skin of a drum is from its frame. So essential is this circumstance, that we find, in cases of paronychia, where the nail is lost for a time, the sensation of touch is materially impaired; being either benumbed or attended with pain, but in both cases comparatively uninstructive, till the regeneration of the nail is sufficiently advanced to restore the cuticle of the affected part to its requisite firmness and stability.

"The rugæ which appear in other parts of the cuticular surface are adapted to the greater mobility of which it is to admit, and to provide against the infinitely varied flexures that the uses of the parts it invests necessarily demand; and they mark out lines, at which a kind of ligamentous structure descends to the coriaceous part of the skin, and which keeps the whole organ steady during these changes, without rigidity; and admits of yielding and softness, without the risk of laceration. (p. 130, 131.)

Next comes the question as to the manner in which perspiration is carried on through the cuticle? how it happens that extravasated blood is absorbed, and not discharged; that the fluid secreted in vesication is not permitted to evaporate, or that various materials pass through it from without? It has always been usual to consider this substance as pierced by innumerable pores: our author, to solve this difficulty, examined portions of cuticle taken from various parts of the body, with a microscope magnifying 140 times, but he failed in detecting any pores; he found instead an infinite number of minute velamina, regularly arranged, of exquisite tenuity, presenting a follicular appearance, and separated from each other by bands of a thicker substance crossing and intersecting them. In these velamina, he conceives the terminal vessels of the cutaneous apparatus to be lodged; and, as long as they retain this connexion, he conceives that they transmit their secretion through them as through a bibulous and exquisitely hygrometrical covering, of the finest delicacy and perfection; and through the same medium, but by tubes taking a contrary direction inwards, absorption is carried on. (p. 135.) Our author adds, that this structure is not without an analogy in the human body; the inhalation and exhalation, constantly going on in the air-cells of the lungs, appearing to take place in a manner almost or precisely similar; and he remarks, that it is in consequence of this structure that vesication on hairy parts, such as the scalp, do not retain the fluid effused so long as others; as, when the cuticle is separated from the hairs, the fluid readily escapes by the perforations they leave.

The fifth Lecture commences with some remarks on the *nails*, which our author thinks may be considered as appertaining to the cuticle; the hair, however, being more properly connected with the cutis. With respect to the nails, we find nothing worth extracting; and a description of the hair would be premature in this place: we therefore proceed to what was formerly called the *rete mucosum*, but which our author has named the *interior epidermis*. It is the seat of the colour of the skin, and is a delicate intermedium between the cuticle and the sensible substance of the true cutis. It is, as well as the cuticle, a bad conductor of heat, and, being placed over a quick one, contributes to the uniformity of temperature. It is capable of being stretched or elongated, by diseased growth, to a considerable degree, and may be regenerated, but only partially so. Our author has never been able to inject this substance; and he is at a loss to account for its blackness in the negro, and its occasional change of colour in the European,—in the areola of the nipples, for example. Its privation from any part of the body is followed by considerable inconvenience; and is, in the case of blisters especially, not always unattended with danger. The interior epidermis is more connected with the sebaceous glands than with the cuticle, as is proved by maceration; and the practical deduction which our author draws from this circumstance, is the necessity of caution with respect to the application of blisters, commonly called *perpetual*, which endanger a destruction of the whole, or of this important part, of the cutaneous apparatus.

Lecture the 6th commences with a view of the fine vascular and nervous fabric situated immediately under the rete mucosum, or interior epidermis; and our author, previous to entering upon this part of his subject, apologizes for not mentioning the labours of those who have gone before him, thinking that such interruptions would rather tend to confuse than elucidate the subject; and this explanation it is necessary to state, lest our readers should be inclined to say that much of the matter we have extracted is any thing but novel: nevertheless, points of novelty will here and there present themselves even to the experienced anatomist and physiologist.

Omitting some general remarks upon the offices of the common integument, we come to a description of the vascular arrangement, which, says our author, is minutely penicillated or villous; a fact long known, but of the importance of which he was not aware until he discovered the velaminous structure of the cuticle, and the conformity of the interior epidermis with it; for, in the cuticular laminæ, thus lined, (he adds,) the penicilli or villi terminate, or are imbedded. This is more especially observable in the lips and ends of the fingers. This structure is supported by a regular and perfect network, very minute, but composed of numerous trunks of vessels for the supply of this ultimate arrangement. All this apparatus is spread upon the corium, and is supplied from large vessels which permeate the corium at acute angles.

Mr. C. hints at, but does not attempt to calculate, the quantity of blood continually flowing over the suface of the body. The uses of this transmission are next mentioned; and our author seems almost diffident in suggesting the interior epidermis as one wide and diffused perspiratory gland, supplied by the above-mentioned vascular texture, and being the medium of conveying that subtile fluid to the cuticle, which thus exhales through its velamina in every degree of gradation. We see no reason why Mr. Chevalier should state so reasonable a supposition in terms so hesitating and humble.

We pass by a page or two containing arguments to prove that the perspiration is not merely a separative process, but one attended with chemical changes, and consequently corroborative of the above views of our author. Besides this destination of the vessels of the skin, they supply nourishment to other parts, such as the nervous papillæ, the pores, the sebaceous glands, and the hairs, then the corium, and finally to the cellular texture.

The papillæ are the next object of consideration, and which, though most evident on the tongue, are assumed to exist over the whole body: they appear to arise from filaments of the cutaneous nerves, and form, *most probably*, in conjunction with the blood-vessels, an essential ingredient in the villous structure of the surface. They are generally of a conical form, and each has an artery appropriated to its own nutrition, and which accompanies it to the termination of its point in the interior epidermis. They are confounded, by Bedlow and others, with the bulbs of the minute hairs or pubescence; but their structure, uses, and their whole arrangement, are entirely different, and their situation only contiguous.

A few prefatory remarks upon the extent and importance of this inquiry ushers in the 7th Lecture; and the first point to which our attention is called is to the pores of the cutis, and which are so obvious when the two epidermides are taken off. Of these pores there are millions on the surface of the body: they are, according to our author, of three kinds,—first, the residences of the bulbs of the hairs; secondly, those of the capilluli, or down, which is, in fact, a congeries of very minute hairs; and, lastly, those of the sebaceous glands. The places of the transit of vessels are but few comparatively, and are readily distinguished by the uncertainty of their arrangement. Our author is of opinion that these pores do not account for either the ordinary or extraordinary perspiration.

The sebaceous glands are next considered, and the remainder of this passage we shall give in our author's words, considering it as a good specimen of the style of the whole work.

"I shall first call your attention to the sebaceous glands; and this because they appear to be first, in order of function, in the growth and destination of the maturing fœtus; in which, toward the approach of the time of its expulsion from the womb of the mother, they are, comparatively at least, more numerous, more constant, and even more necessary, and more employed in the economy of the animal, than when it has emerged from its prison, and taken upon itself independence of life and of activity. Incapable of respiration, the perspiratory office not being begun, neither urinary nor alvine excretions calling for discharge, consciousness not yet awake, the senses only in a state of preparation, and even the muscular energies only such as to give confirmation of its vitality, and of its being on the approach to fitness for its future condition; it chiefly requires a supply of nourishment and warmth, which it derives from its parent, to ensure its growth from within; and of a safeguard exteriorly, to render all this compatible with the necessary conditions of its temporary abode, and its approaching departure from it. Exteriorly, therefore, it is surrounded by the liquor amnii; which affords a gentle and equable support to the growing fabric, and protects it from many risks of injury from violence *ab extra*, during its early and pulpy state; and, at a more advanced period, seems destined at once to impede perspiration from the skin, and to prepare and keep fit that very skin for that very office, when it shall arrive into the more rare atmosphere in which it is to live. But, in order to secure the cuticular covering of the skin from harm, by being so long macerated, as it were, in the fluid around it, these glands secrete a clammy and somewhat unctuous substance, repulsive of water, which is entangled and rendered adherent to it by a soft and minute pubescence, composed of millions of capilluli, or little hairs, the roots of which rest upon, or rather in, the corium; they then pass obliquely through both the interior and exterior epidermis to the surface, to fulfil their offices. And the separation of these leaves the appearance of pores." (p. 182, 183.)

Of these glands there are two sets, one of which Mr. Chevalier believes to have escaped notice hitherto: they are very minute, and lie between the second epidermis and cuticle, to which they appear firmly attached. Our author at one time considered them as appropriated to perspiration; but closer inspection convinced him that this was not their office, since they were not to be found where perspiration goes on evidently and copiously, but where the sebaceous secretion is less called for; besides which, they are not reproduced when destroyed; and, although both the epidermides are so, yet perspiration still goes on. The deeper-seated set of these glands reside behind the inner epidermis, nourished and influenced by the reticular and villous texture of blood-vessels and nerves, and attached to the coriaceous part of the integument beneath them. The secretion from these glands, and the uses for which it is destined, are too well known to require description: we therefore proceed to give some account of the hairs and the pubescence, or down, which, like the former, grow from small bulbs, and are imbedded in the corium, supplied by vessels from the reticular plexuses; they then pass through both the epidermides at very acute angles, closely embraced by the cuticle, which does not allow them to be easily detached: it is therefore obvious that the capillary perforations cannot yield the perspiration, for the obliquity of their course would oppose an almost insurmountable obstacle to its transmission; or, rather, it constitutes a perfectly valvular obstruction.

Omitting some remarks on the formation of the hair in man and other animals, together with some speculations on the probable share which the skin has in the oxygenation of the blood, a subject formerly touched upon by Mr. Abernethy; and the more obvious and commonly observed sympathy between the skin and the whole intestinal canal, we come to the 8th Lecture, more immediately devoted to the *corium*, the basis on which the parts above mentioned are spread out. It is described as a fibrous structure, interwoven with each other in various directions; it is less mutable, and less easily regenerated, than the rest of the integument; and, when it is re-produced after entire destruction, it contains neither capillary nor sebaceous pores. Our author mentions next its elasticity, its wonderful adaptations under various circumstances of disease, and notices the two distinct substances of which it is composed,—namely, the fibrous and the pulpy. He is also inclined to think that it possesses some muscular power, analogous to that of the pallium of the molluscæ, or the chrystalline lens; and he has found that, after maceration, like muscle, it is sooner changed into adipocere than many other parts of the body. This, as a reason, we confess, appears to us not to amount to much, because our author himself acknowledges that most other portions of the human frame are subject to the same change.

The ninth Lecture commences with a description of the *tela cellulosa*, and which is carried on through several pages, without, however, affording any thing particularly novel or striking, either in the anatomical investigation, or the pathological remarks which they give rise to; and the lecture concludes with a neat summing-up of all the principal points which our author has dwelt upon in the course of his investigations respecting the skin, but which we need not repeat, as, in truth, we have endeavoured to make this article an epitome of the work itself.

Subjoined there are a few pages relative to the present state of surgery, and the failure of the application made a few years ago to Parliament for some power to control unauthorised practitioners. This is a subject upon which we feel that much might be said, but we forbear from giving expression to those feelings, principally from the respect that we entertain towards Mr. Chevalier, who, we are confident, has the respectability and character of the profession sincerely at heart; but we must be permitted to observe that we (and we believe that we are only speaking the sentiments of the majority of the profession,) should feel serious apprehensions of the powers of the College, constituted as that College is, being increased in any degree. Of the governing members generally, we entertain a high opinion, and for some of them individually a sincere regard; but the system is radically bad, as every system must be, which places the most distinguished talent at the mercy of a very limited number of persons. We very much doubt, indeed, whether any enactment of the legislature could prevent the evils complained of; for, in fact, it is the number of dupes that makes the quack; and many of the practitioners of whom our author complains are, we are sorry to say, not unauthorised; most of them have passed the ordeal of the College, and their deficiencies are not those of the head, but of the moral sense: their motto appears to be—

———————"rem

Si possis recte; si non quocunque modo rem."

---

**Art. II.**—*Pharmacopœia Collegii Regalis Medicorum Londinensis* MDCCCXXIV.—Londini: Longman, 1824. pp. 205.

**Art. III.**—*The same, translated into English.* By Sir George Leman Tuthill, Knt. M.D. F.R.S. &c.—Longman, 1824. pp. 205.

It is, as nearly as we can recollect, about two years since we first heard that the Royal College of Physicians of London were engaged in revising their Pharmacopœia; and, in common with many of our professional brethren, we were not a little anxious to see the fruit of their long, and doubtless well digested, labours. Of the propriety, and indeed we may say of the urgent necessity, for such a revisal, we were quite as much convinced as the Fellows of the College themselves. We had never doubted for a moment about the paramount importance of a good national Pharmacopœia, and we felt that the united labours of *all* the members of the College could not be better employed than in improving a work, which, as his Majesty's proclamation most truly states, "is intended for the Common Good of His People, wherein the Lives and Health of His Majesty's Subjects are highly concerned, and which, by preventing all deceits, differences, and uncertainties, in making or compounding of Medicines," must necessarily "contribute to the public good of his Majesty's subjects." We never, it is true, heard that any Licentiates of the College were applied to, to give their advice and assistance in correcting and reforming a work of such vast importance to *them*, as well as to the Fellows; but this appeared to us a matter of small consequence, when we reflected that there are at present eighty-eight Fellows of the College, besides three candidates and five inceptor candidates, many of whom we know to be most excellent physicians, well versed in the principles of pharmacy, and calculated in every respect to produce a work of public utility; and such, too, as might reflect credit upon the body from which it springs, on the country to which it belongs, and on the age in which it appears. Without wishing by the selection to depreciate, in the slightest degree, the merit of many most scientific and highly-respected members of the College, we may be permitted to add, that we looked forward with some sort of national pride to the labours of Dr. PARIS, in the important work which the College had taken in hand. This gentleman had been received into their body since the period when the last important changes had been made, (1809.) His excellent work, entitled Pharmacologia, which we have no doubt all our readers are well acquainted with, prepared *us*, as it must have done *them*, for some really valuable additions to the standard volume of English pharmacy; and his official situation of professor of materia medica to the College, could not fail to have placed him in circumstances peculiarly favourable to render his services efficient. We felt, moreover, that the late edition of the London Pharmacopœia really did require revision, and that it was not mere caprice, or the love of change, which prompted the influential men in the College to recommend such a measure. We knew, for instance, that a great many drugs had a place in the pages of that book, which, never being prescribed, no apothecary ever thought of keeping, and the expulsion of which, therefore, would have been creditable to the College. We were fully sensible, too, that a great many "*oils and waters had been distilled, and other extracts made*," according to processes "not directed, prescribed, and set down in the former edition of the said book," (and that, too, for very weighty reasons;) and we could not but acknowledge it as a very desirable thing, that the actual "manner and form" of compounding medicines should correspond *in some degree* with the mode prescribed and directed by the College, knowing, as we did, "that offenders to the contrary not only incurred his Majesty's just displeasure, but were liable to be proceeded against for such their contempt and offence, according to the utmost severity of law." Lastly, in forming our estimate of the probable value of the forthcoming edition of the Pharmacopœia, we remembered how much assistance has been voluntarily offered of late years, in the shape of criticisms upon the edition of 1809; and we were confident that the Fellows of the College, by their adoption of the most valuable of the suggestions thus thrown out, would have exhibited, in their corporate capacity, the same liberality of sentiment which characterises them as individuals.

Such were the predominant feelings in our minds when we heard it confidently rumoured that a revised edition of the Pharmacopœia was speedily to appear; and it was with no common or pretended anxiety that, on the 14th of February, we first found it on our bookseller's counter. Perhaps we had formed exaggerated expectations; but we are sure, at least, that, if we did, multitudes of our professional brethren in London shared in the same very natural, and we hope easily to be forgiven, error. We strongly suspect, too, that a very large proportion of our country readers will have experienced a similar feeling, and that when they see announced, on the wrapper of this Number, a Review of the "Pharmacopœia Collegii Regalis Medicorum Londinensis MDCCCXXIV." their paper-folders will be very speedily put in requisition. Let them, therefore, participate in our surprise, when we inform them that the changes which have been made are, at first sight, hardly discernible; that a moderate list of *errata* would easily convert the Old into the New Pharmacopœia; and (tremble Messrs. Longman and Co. while we say it!) that any of our readers, who may not happen to have seven shillings to spare, may, actually by a little attention to the next two or three pages, save himself the expence of a copy of the new edition.

At the Court at Brighton, on the 19th January, 1824, present, the King's most excellent Majesty, Lord Liverpool, his Grace the Duke of Wellington, and eight other members of the Privy Council, Sir Henry Halford presented a humble memorial, setting forth that the President and Fellows of the College had, "*with great care, pains, and industry*," revised, corrected, and reformed their Pharmacopœia. The *reform*, however, turns out not to be very *radical*; and, though we are well persuaded that the President and Fellows spared no pains or care in revising their book, yet it seems to us that it would have been only a proper compliment to the framers of the last Pharmacopœia, if they had added—in the very few places in which it appears to us that it requires such revision, correction, and reformation. But our readers are doubtless anxious, by this time, to be made acquainted with the changes which have taken place; and we shall therefore proceed, without further preface, to the *dry detail.*

In the first division of the Pharmacopœia, viz. the Materia Medica, the alterations are as follow:—Two drugs are thrown out, viz. Aloe Vulgaris, and Vinum Album Hispanicum (Anglice *sherry*); six new simples supply their place, viz. Cubebs Pepper, Elecampane Root (Inula Helenium), the Garden Lettuce (Lactuca Sativa), the seeds and leaves of Stramonium, the Croton Oil, and Rhatany Root (Krameria Triandra). Besides which, admission has been given to the *seeds* of the Conium, Colchicum, and Digitalis; the Pyrolignic Acid has been introduced, under the title of Acidum Aceticum Fortius, (a term decidedly objectionable, as it has been already appropriated by the Edinburgh College to that stronger variety of acetic acid which is obtained from the superacetate of lead;) and, lastly, permission has been given by the College to do that, which, for many years past, apothecaries have done without permission,—we mean, purchase their crystals of citric acid in the market, instead of making it themselves. Among the drugs obtained from the mineral kingdom, the changes are even still fewer. Bismuth is admitted; the Sulphate of Potash, and the *Subcarbonate* of Magnesia (as it is now called), are allowed a place here as well as among the Præparata et Composita; and the Glass of Antimony is restored, under its old designation of Vitrum Antimonii. Let the chemists dispute till they are tired about sulphurets and hydro-sulphurets, chlorides, and muriates, oxyds, and acids; and let the appended chemical explanations (in the second column of the catalogue) vary according to the progress of science, or the prevalent doctrines of the day; but let the designations of medicines continue unaltered. On this principle, we subscribe to the propriety of changing Arsenici Oxydum to Arsenicum Album, Lytta to Cantharis, and Resina Nigra (common pitch) to Pix Nigra. The following notices complete the changes which this department of the Pharmacopœia has undergone:—Burgundy pitch (the Pix Arida of the last edition,) is now Pix Abietina, but destined, we will hope, hereafter to revert to its old and well-understood denomination of Pix Burgundi. Calumba is announced, on the authority of De Candolle, to be root of the Cocculus Palmatus; Kino as an extract from the Pterocarpus Erinacea; and the tree which affords Cardamoms, (the Elettaria Cardamomum of the last Pharmacopœia, the Amimum Repens of the Edinburgh College,) has received another *alias*: it is now the Matonia Cardamomum, so called in compliment to one of the learned members of the College;—and this is all, unless, indeed, any of our readers should be singularly precise, and such may like to know that the term *Marmor Album* is now substituted for Lapis Calcareus.

We now come to the *Præparata et Composita.* In the first division, Acida, we have to notice the term Acidum Aceticum *Dilutum*, as applied to distilled vinegar, and the introduction of the common process for preparing Tartaric Acid. In the *Alkalia et Eorum Sales,* the only change which has been made is unquestionably a good one; that is to say, the Carbonates of Soda and Potash are now directed to be made by saturating solutions of the respective subcarbonates with carbonic acid gas.

The process for making Emetic Tartar is simplified; and we believe this to be one of the best alterations which the College have made. The glass of antimony, finely powdered, and cream of tartar, are directed to be mixed, and boiled for a quarter of an hour in distilled water, and the solution evaporated till crystals begin to form. Nothing, certainly, can be more elegant than this. Of the Antimonial and Steel Wine we shall speak presently; in the mean time, it may be curious to notice that, of the three hundred and forty formulæ, which the last edition of the Pharmacopœia contained, *one only* has been expelled! One only could find no friend among the eighty-and-eight fellows of the College; and that one is the Oxydum Antimonii. Under the *peculiar* circumstances of this degradation, we wish we could say any thing in favour of the medicine which we have lost; but, with all a reviewer's disposition to cavil, we must confess that, if one only was doomed to suffer, the sentence has fallen upon the true offender.

A Subnitrate of Bismuth is introduced, upon whose suggestion, or with what view, we have no means of judging; as Sir George Tuthill's translation is without notes. A great improvement has been effected in the process for preparing Calomel. Corrosive Sublimate does not enter at all into any part of the process, the additional quantity of mercury being added *in the first instance*. This process has been followed for many years at Apothecaries' Hall, and was, we believe, originally suggested by Mr. William Brande. A change has likewise taken place in the formula for preparing the Oxyd of Zinc. It is now ordered to be made in the *humid* way; a solution of the sulphate of zinc being decomposed by the volatile alkali.

The distilled waters of Cinnamon and of the three Mints may be made, either according to the old process, or by distilling from the essential oils, or, in the case of the mints, from the fresh herb, at the option of the apothecary. Three of the old infusions have received the additional appellation of *compositum*. In so far as the Infusum Lini and the Infusum Sennæ are concerned, we can see no great objection, though we cannot help thinking that the term Infusum Sennæ *Aromaticum* would have been preferable. But on what principle the College have chosen to call the common infusion of roses *Infusum Rosæ Compositum*, we are quite at a loss to comprehend. So far as we can judge, it is not a compound, but a very simple infusion; and, if the College wished to specify that it was acidulous, the term *Infusum Rosæ Acidum* might have been adopted, with the merit of being equally classical, and somewhat more appropriate.

To the list of *Extracts*, the College have added those of Lettuce and of Stramonium Seeds. The Spirits of Rosemary,

Cinnamon, and of the three Mints, are now directed to be prepared from the respective essential oils, instead of the herb and bark as heretofore.

In the class of *Tinctures,* the single change which has been made is employing rectified instead of proof spirit, in the Tincture of Ginger. This is decidedly an improvement; but it is more than counterbalanced by the extraordinary, and, as it appears to us, the most uncalled-for, changes in the class of *Vina.* Our readers will, perhaps, hardly credit us, when we tell them that, though a dilute spirit is now employed instead of wine, the term Vinum is still continued, not only in the case of those preparations which have long been known under the name of wines, (such as the Vinum Ferri and Vinum Aloes,) but even in the newly-introduced preparation, the Vinum Colchici. Singular as this is, it falls far short of the mode in which the College have treated the nomenclature of antimonial wine. In the last Pharmacopœia, when this medicine was really made with sherry, and when the College might with perfect propriety have called it Vinum Antimonii Tartarizati, they called it, as our readers well know, *liquor.* Now, when they have ordered it to be made with a very dilute spirit, and when they might with perfect propriety call it Liquor Antimonii Tartarizati, they return to the old name of *vinum.* This is a sort of *hide and seek* way of changing names, which appears to us altogether unworthy of the Royal College of Physicians of London. The very general use of colchicum wine naturally suggested to the College the propriety of introducing such a preparation; but the College process furnishes us with a dilute tincture, and we are by no means prepared to say that its effects will correspond with those of a vinous infusion of colchicum. The College have adopted the formula in use at St. George's Hospital, without, as we imagine, much consideration. The quantity of colchicum root ordered is so large, that the fluid barely covers it; and, if the root happens to have become a little dry by keeping, more than one-half of the spirit and water is absorbed. We are, in fact, totally at a loss to understand by what principles the College have been guided in directing the proportions for their two officinal preparations of the colchicum root. In the vinum colchici, one ounce of the sliced bulb is macerated in one fluid ounce of weak spirit; in the acetum colchici, the same quantity is macerated in sixteen fluid ounces of distilled vinegar. Surely the College must be wrong either in the one or the other. We omitted, by the way, under the head of Spirits, to introduce to our readers a new preparation of the seeds of colchicum, called the *Spiritus Colchici Ammoniatus,* made by digesting an ounce of those seeds in half a pint of Spt. Ammon. Aromat. The precise object of this preparation, in the absence of all explanatory statements by the translator, we are, of course, unable to lay before our readers.

To the old officinal *Syrups* is now added a simple syrup of Sarsaparilla; and the *Confections* have received into their ranks, (under the title of Confectio Piperis Composita,) Ward's paste for piles. It is obviously for the sake of this medicine that elecampane root has again been admitted to a share of the College honours. When we add to this, that Plummer's pill is now directed to be made of a proper consistence by means of spirit instead of mucilage, we have mentioned every change which the Pharmacopœia has undergone from the "care, pains, and industry, of the College or Commonalty of the Faculty of Physic in London."

That these changes are not such as will satisfy the expectations of the profession, we feel very little hesitation in avowing. Rumours have reached us that the *first specimen* of a new Pharmacopœia, which was circulated among the Fellows of the College, was such in *deed* as well as name; that Quinina and Prussic Acid, and Iodine, &c. &c. &c. were therein to be met with; and that changes were contemplated of no ordinary magnitude. We are not, indeed, among the number of those who would blame the College for not introducing all the new French remedies, as they have been called. On the contrary, we think it highly to their credit that they have refused to stamp a degree of character upon these drugs, until experience has more fully established their claim to such a privilege. But, though we do not blame the College for these sins of omission, we cannot say we are satisfied when we find them retaining so large a number of inert and useless drugs, and neglecting many obvious improvements in their pharmaceutical processes. With the exception, indeed, of the saline and metallic preparations, we think the present in no degree superior to the last edition of the Pharmacopœia. Who is there, for instance, who ever prescribes, or has any wish to see retained, such drugs as the following,—Lauri Folia, Rumex Acetosa, Oxalis Acetosella, Allium Sativum, Centaurii Cacumina, Dauci Seminæ, Helleborus fœtidus, Fucus Vesiculosus, Menzanthes trifoliata, Petroleum, Allium Porrum, or even Rubia Tinctorum?

We do not wish to be hypercritical, but we should like to be informed what is gained by retaining in the catalogue of the Materia Medica, the inversions of the names of drugs? as, for instance—

| | |
|---|---|
| Antimonii Sulphuretum, | Sulphuretum Antimonii, |
| Cupri Sulphas, | Sulphas Cupri, |
| Plumbi Subcarbonas, | Subcarbonas Plumbi, |
| Sodæ Subboras, | Subboras Sodæ, |
| Sodæ Sulphas, | Sulphas Sodæ. |

If the College think it of importance to change Elaterii Poma into Elaterii Pepones, surely these inversions might have been spared, with at least equal propriety. To do the College justice, however, it should be added, that the explanation of Zincum, which was given in the last Pharmacopœia, is expunged in this.

While we see the Fellows of the College, indeed, engaged in such trifling alterations as that of *Zinci Frustulorum* for *Zinci in frustula fracti*, we cannot avoid thinking they would have been equally well employed had they directed their attention to the processes by which their decoctions, tinctures, and syrups, are made. Had they done so, they would probably have found reason to think with us, that the Decoctum Aloes Compositum contains twice, if not three times, as much compound tincture of cardamoms as there is any occasion for; that, if the College are so scrupulous as to call the infusion of roses *compound*, because it contains a little acid, the Tinctura Rhei might have received some appellation (such as Tinctura Rhei Aromatica,) to let us know that it contains such a quantity of cardamom seeds and saffron as in a considerable degree to hide the taste of the rhubarb, and to make it a pleasant cordial; likewise that, in the simple tincture of rhubarb, the quantity of spirit ordered is just double to what is directed for the compound tincture, though the proportion of rhubarb is the same; proving, we think, pretty clearly that either the one or the other of the processes must be defective. A little attention to the same department of the Pharmacopœia would also have shown them that the syrup of orange-peel is ordered to be made with at least one pound of sugar more than is necessary; that the syrup of senna contains double its proper quantity of manna; and that the Syrupus Papaveris would have been greatly improved by ordering a given proportion of the extract to be rubbed down with simple syrup.

We know no good reason for retaining the Vinum Veratri. We cannot avoid thinking that a little of the "care, pains, and industry" of the College might have been advantageously employed in revising the process for preparing the Extractum Elaterii, and the compound Decoction of Sarsaparilla, where the sassafras is still directed to be boiled for a quarter of an hour, instead of being *infused* at the termination of the process.

But we are pressed for time, and can no longer continue our criticisms. We have only to add, that the College have not thought it necessary to write a new preface, any more than Sir G. Tuthill to add any explanatory notes. Of this translation, indeed, we do not know that we can say any thing better than what we have just read in Mr. Longman's advertisement,—viz. "that it is printed page for page with the original Latin, and that both may be had done up together."

### DIVISION II.

### FOREIGN.

ART. III.—*De Nervi Sympathetici Humani, fabrica, usu, et morbis; Commentatio Anatomico-Physiologico-Pathologica, &c. &c.* Auctore J. F. LOBSTEIN, &c.—Parisiis, 1823.

[Continued from page 165.]

THE development of this nerve in the fœtus has likewise occupied the attention of this industrious anatomist. He found the tunic of the nerve sufficiently distinct at the age of fourteen weeks, when the embryo was three inches in length. In the chest it was rather thick and reddish, from the thoracic ganglia being still near to each other. The superior cervical ganglion was well marked, being two and a half lines in length, and half a line in thickness. The splanchnic nerve was formed of a very fine thread; the semilunar ganglia were very obscure.—In a small embryo of five months, the trunk of the sympathetic nerve was very distinct, forming a continuous thread, which extended without interruption from the head to the pelvis. The gradual evolution of the nerve, with the progressive growth of the other parts, is detailed with extreme minuteness and apparent fidelity; but into these we cannot enter. In old age, the ganglia become paler and less pulpy than in youth: this was particularly conspicuous in an old man, aged eighty-four, in whom the branches given off by the ganglia appeared less numerous: the renal plexus, for example, was constituted by a few nervous twigs, and these were drier than usual, and more faded. LUÆ has made the same observation.*

The *internal structure* of the sympathetic nerve occupies the fourth chapter, which is one of much interest: the subject is considered under the several heads of trunks, branches, ganglia, and plexuses. By the trunk is understood that cord extending from the head to the pelvis, situated near the bodies of the vertebræ, studded with ganglia, and sending off one set of branches communicating with the spinal nerves, and others which go to the different organs; that, in short, which was universally held to be the trunk until Bichat, making facts bend to theory, acknowledged it only as a nervous thread, connecting one ganglion with another, so that the number of communicating branches equalled that of the ganglia. Notwithstanding its ingenuity, and the many proselytes it has gained, there are few physiological doctrines which appear to us to have obtained so much reputation with so little merit, as the ganglionic

* *Comment. de Nervis Arterias Venasque comitantibus. Comment. Anat.* Gotting. 1786.

system of M. Bichat: we are, therefore, gratified to find our own humble judgment backed by the powerful authority of so eminent a man as Lobstein. His reasons for dissenting from the opinion of Bichat are simple, and, if we admit the accuracy of his anatomical details, must likewise be regarded as satisfactory. He informs us, that, during twenty-four years' application to the study of anatomy, (and that the application has been assiduous his writings bear witness,) he never remembers to have seen one single example in which the sympathetic nerve has been interrupted, either in the thorax or elsewhere, although he has seen its direction changed before its entrance into the abdomen, so that he might have supposed it had ceased, unless he had instituted a minute and careful examination. He has attentively examined the trunk of the nerve, from its exit from the first cervical ganglion unto the fifth thoracic ganglion, by an analysis, effected with the assistance of maceration; and he states that he clearly saw this same trunk pass through the middle and third cervical ganglion, arrive at the first thoracic, mingle there with many filaments, pass out again, be received into the second thoracic, and occupy its inner side; thence proceed to the third and fourth; lastly, cohere with other filaments, or these always occupying the internal side of the ganglion. In this manner he has traced the nerve to the second sacral ganglion; and hence infers that the fine threads forming the trunk of the nerve pass through the ganglia in an uninterrupted course, "sed tenerrema filamenta quæ truncum componunt, ganglia trajiciunt, tramite non interscisso." Besides, the trunk of this nerve, when minutely examined, is like that of others, (the median, for example, or the cerebral,)—viz. composed of threads, which, placed in juxtaposition, are too much interwoven to admit of being separated with the knife.

With respect to the branches: Bichat, it will be remembered, held these to be of two kinds;—one set like the cerebral nerves in colour and composition, being constituted of medulla enclosed by neurelima; and another, (those, namely, which go off from the ganglia,) soft, fine, of grey colour, having an indeterminate structure composed of medulla and neurelima, not disposed as in the preceding case. This opinion is combated by SCARPA;* and it is enough for us to add at present, that it is likewise opposed by the author before us.

In examining the ganglia, Lobstein fixed them upon pieces of wood, daily softening them with fresh limpid water, till he reduced them into downy globules, in which he could trace the relation of the most minute fibres of the nerves; an object which was further assisted by placing them on plates

* *Annotat. Academ.*

of ebony, by which dark ground the white filaments were rendered more distinct. He has given two plates, executed on this plan. The difference in the structure of the ganglia, which he thus discovered, we have hinted at above. Scarpa has spoken of a gelatinous matter which exists in the ganglia; and, indeed, a general idea has prevailed that the substance of these bodies bears more or less resemblance to that of the brain. Our author has found this jelly in all ganglia, but more particularly in those of hydropic patients; but the matter has never resembled oil or fat. It passes into the branches of the ganglia, both external and internal, so that sometimes they assume a diaphonous appearance in the fœtus, and even in adults who are young. Notwithstanding this, however, Lobstein does not seem inclined to regard it as a certain and constant phenomenon, properly referable to the natural state of the ganglia. On the contrary, it very rarely occurs in the thoracic ganglia, or in the cardiac and pulmonary plexuses: it is more frequent in the neck, especially in the nerves constituting the carotid plexus, which likewise retain their softness the longest.

Although he unequivocally denies any resemblance in structure between the brain and the ganglia, he admits, nevertheless, the existence of two kinds of substance in these bodies. In the smaller ganglia, the interlacing of the filaments can be more effectually untwined, and, on applying a microscope, the linear direction and fibrous appearance sufficiently distinguish the characteristic form of the nerves. Adjacent to this, however, is perceived another substance, grey, flocculent, and of an orbicular figure, which cannot be traced into any plexus, and which constitutes the second material of which the ganglia are composed. As a general rule, it appears that the larger the ganglia are, the less they have of this grey-coloured globular substance; but the first cervical is the only one in which this distinction of component textures may not be detected. This ganglion was subjected for thirty days to maceration: it was softened, indeed, but long remained a homogeneous mass. Examined through the microscope, it presented the appearance of innumerable fine threads, not unlike cotton, but could not by any means be divided into fasciculi. Of the vessels of the ganglia, it is enough to say that they are provided with arteries, veins, and lymphatics.

### *Section Second—Physiological.*

We now come to the second great division adopted by M. Lobstein in his account of the sympathetic nerve,—viz. that which relates to its physiology; and, before we enter into the particular views of our author, it may not be amiss to take the opportunity afforded by the work before us, of recalling very briefly to our readers the opinions entertained on this subject by others.

So long ago as the time of WILLIS, the sympathetic was regarded as executing functions of a nature distinct from those of other nerves, and was stated by him to be intermediate between the conceptions of the brain and affections of the precardia; and, again, to communicate between the actions and passions in almost all parts of the body; the ganglia being supposed to afford receptacles for the animal spirits. This doctrine, it is to be observed, does not differ essentially from that of more modern date. VIEUSSENS adopted opinions nearly similar, adding a fermentive process in the ganglia, which gave rise to muscular contractions.* According to WINSLOW, the ganglia are nuclei, or separate origins, of the sympathetic nerves, resembling little brains: this distinguished anatomist was the first who applied the name of *sympathetic* to this nerve; and he denies that it begins in the carotic canal, but states, on the contrary, that it sends branches back into it, for the purpose of communicating with the fifth and sixth nerves. MECKEL makes the use of the ganglia to be, to divide the branches into smaller twigs, and then into filaments; to enable these twigs to arrive at their destiny by various directions; and, lastly, to unite many branches in one trunk.† JOHNSTON regarded the ganglia as little brains, composed of two substances,—viz. grey and medullary; and in which, besides the nutritious vessels, nervous fibres are present, which lose their rectilinear direction, and receive the new constitution of the parts. These ganglia were supposed by him to evolve nervous energy, as well as the brain, and communicate it to the organs. He held the ganglia, in short, to be the origins and fountains of the nerves belonging to the involuntary organs; by their aid, the motion of the heart, intestines, and other parts beyond the control of the will, was supposed to be carried on. SCARPA, adopting the opinion of Meckel and Linn, gave to the ganglia a three-fold use,—disuniting, intermingling, and again binding up in new sets: "nervos nempe disjungendi, commiscendi, ac iterum colligandi." This author endeavours to show that the ganglia are so placed as to send off, in smallest possible space, and with wonderful facility, branches of nerves to the contents both of the thorax and abdomen: thus, taking the splanchnic, he would fain demonstrate that its origin is derived *first* from threads of the intercostal, and perhaps also of many spinal nerves; *secondly*, from filaments of some of the dorsal nerves going from the spine to the thoracic ganglion; and, *lastly*, from fibres of

* *Neurograph. Universalis*, lib. iii. cap. v.

† *Memoires de Berlin.*

the fifth and sixth effecting a communication with the brain. Lobstein naturally remarks, "Quam altissima hujus nervi origo!" Scarpa concludes by giving it as his opinion, that all the viscera supplied from the ganglia of the sympathetic receives nerves composed of filaments from many sources; some derived from the brain, and others from the spinal cord. Bichat adopted and improved upon the ideas of Winslow and Johnston: according to him, the system of nerves which is distributed on the organic life is nothing but a congeries of many centres, from whence the nervous energy is transmitted to the organs. The anatomical difference between the nerves of organic and animal life is made to consist in this, that the latter have only one source,—viz. the brain, while the former have many,—viz. the ganglia; to that the great sympathetic is endowed with as many distinct centres as it has ganglia. These are joined by what others have regarded as the main trunk, but what Bichat holds to be merely anastomosing twigs. Reil, an anatomist of great industry and talent, embellished still further the doctrine of Bichat, and constructed upon it a theoretical system. According to him,* the sympathetic constitutes a peculiar nervous system, distinct from the brain, and called the *ganglionic.* This system alone occurs in some orders of animals; it alone supplies the organs of nutrition, by which the wasted powers of life are renewed,—and hence the name of *vegetative,* sometimes applied to it. It is found in the lowest scale of animal existence, beginning with the moluscæ, and gradually ascending to man. In the more perfect species, it is connected with the cerebral, or *animal* system of nerves; but never, according to Reil, originates or begins in it. The nervous system of the brain differs from that of the ganglia in this, that its branches converge from the different parts towards the head, having their roots, as it were, implanted in it: the brain, therefore, is the only centre of this system. The ganglionic, again, acknowledges no particular centre, but is general; having no common focus of action, but holding the various organs in its extensive sympathies. This it does by accompanying the arteries into the parts, by sending off conductors to communicate with the brain and spinal marrow: thus, the renal nerve and both splanchnics are supposed to be conductors; but, as this chain of communication would transmit to the brain the operations going on in the internal organs, and likewise place these under its control, so the ganglia are necessary to interrupt this intercourse. Thus, for example, if there was no cervical or thoracic ganglia, if the cardiac nerves proceeded directly from the brain and

---

* *Archiv für die Physiologie.*—The profession is indebted to Mr. H. Mayo for a condensed translation of some of Reil's Essays.

spinal marrow, the heart could be stopped, like any other muscle, by the influence of the will: the ganglia, in short, according to this view, are, in the operations of the animal frame, what irritating bodies are in physical experiments. The ganglionic system receives impressions, indeed, and communicates them; but these occurrences take place only within its own territory, and are not, at least in the healthy state, transmitted to the sensorium. But, in disease, matters are changed; and those bodies, which were before non-conductors, now become conductors, and transmit the sensations.

Some of the most recent theories respecting the use of the sympathetic nerve are those of M. Broussais:* he, like others who have gone before him, acknowledges the sympathetic nerve as a peculiar system, forming a centre of sensation proper to itself, which not only transmits sensations to the sensorium commune, by which the actions of the viscera are excited, but likewise produces influences in it, which are transmitted by the cerebral and spinal nerves to the muscles of voluntary motion. In the fœtus, he supposes the sympathetic alone to be effective, presiding over the nutritive and secreting functions, giving energy to the heart, and sometimes carrying its influence even to the brain itself; and thus exciting those muscular motions which the fœtus in utero is known to perform. Acephalous fœtuses, wanting alike the brain and spinal marrow, likewise are capable of movements; and this is supposed to take place in consequence of the vital energy of those branches of the sympathetic which join by anastomosis with the spinal nerves. In the human being, after birth, this nerve, mediating between the sensations of internal viscera and the brain, keeps up those numerous sympathies which are illustrated by innumerable phenomena.

We now come to inquire into the opinions of Lobstein on this subject, and we trust that the importance and interest of the question will be apology for the space which we have devoted to it. The author, after some general observations, divides his remarks into three heads, considering, first, the power or energy which the sympathetic nerve possesses; secondly, the functions which it performs; and, thirdly, the mode or mechanism by which such powers are excited, and such functions performed.

With respect to the first of these, M. Lobstein agrees with most authors that the various divisions of the sympathetic are endowed with the same general properties of other nerves; that is, that they are the fountains of vital energy, whence proceed the tone and healthy condition of the viscera. It is con-

* *Reflexions sur les Fonctions du Système Nerveux en general, &c.—(Journal des Sciences Medicales,* tome xii.)

jectured that the ganglia may act as a sort of laboratory for eliminating those principles which are communicated to the organs: at least, our author rejects the mechanical explanation of those who attribute to these bodies the office of interlacing, and again distributing the nervous twigs; and, in confirmation of this idea, we are referred to the first part of the essay, in which it is mentioned that, besides the threads of nerves, there existed in the ganglia another substance, soft and flocculent, and having no fibrous appearance. As the most numerous branches of the sympathetic belong to the arteries, embracing and accompanying them even into the organs, and terminating in their external coats; from which it follows that these vessels are more immediately under the control of the nerves, and supplied by them with the power they exercise in effecting secretion and nutrition. The nervous energy is further supposed to be diffused by many of the cellular tissue, in which the minute ramifications are lost, and of which we have already spoken. Physiologists, anxious to ascertain to what extent the sway of this system of nerves pervaded the organs which it supplies, have instituted numerous experiments; Lobstein has followed the example set by others, and some of the results are interesting and important. He killed various animals, particularly dogs and cats, soon after birth, by wounding the spinal marrow, taking care that they should really be dead before his experiments were begun. He then tried to excite the branches of the sympathetic, but did not succeed in communicating any motion to the organs which they supplied. His experiments were next directed to the sympathetic nerve and par vagum, in the region of the neck. On applying galvanism, no movements were excited in the heart, stomach, or intestines: it is true the heart contracted, and the peristaltic motion went on; but these operations were nowise dependent upon the influence of the foreign stimulus. The same fact of the organs refusing to be artificially excited to movement through the sympathetic nerve, was further illustrated by the following occurrence:—During a severe and difficult labour, a fracture of the calvarium took place, and the brain escaped. The muscles contracted, and the heart trembled by the simple contact of the air; but, on arming the sympathetic, and applying galvanism in the usual manner, no phenomena occurred in those organs which, from their structure, were capable of moving; while, on the other hand, powerful muscular contractions were excited on applying the same stimulus to the cerebral nerves. These experiments, it is to be observed, do not, and cannot, apply to the function of secretion, which is not manifested by any perceptible movement; and, with respect to the absence of movement in the heart, &c. it is contrary to the results obtained by others; and it may be regarded as established, that the galvanic influence does stimulate the viscera, when communicated through a branch of the ganglionic, as well as the cerebral system. It is thus concluded by Lobstein that no essential difference exists between them. In the non-vertebrated animals, and in some orders of the vertebratici, sometimes the sympathetic, and sometimes the par vagum, is deficient; but observing this rule, that the one which is present supplies the place of that which is absent: thus, we seen ganglionic and cerebral nerve becoming substitutes for each other. In addition to this, we have the voluntary nerves converted into involuntary, and *vice versa*. In conclusion, the powers of all nerves are believed to be the same; and the varieties of the phenomena of life to depend upon the differences of the stimuli proper and peculiar to each system.

With respect to the functions, the sympathetic, in the first place, is to be regarded as presiding over nutrition, in its extensive signification. This, among other experiments, is proved by those of M. Dupuy, who cut out the cervical ganglia of either side in horses: among phenomena the most remarkable was the wasting of the whole body and swelling of the feet.* Independent of analogical reason, we have direct proof, in the experiments of Mr. Brodie, that this system is likewise necessary to secretion. This distinguished physiologist injected a solution of arsenic, which has a remarkable tendency to promote the flow of gastric juice: he divided the par vagum and sympathetic, the consequence of which was an interruption of the usual secretion. The influence of this system on the heart and arteries is almost too obvious to need any illustration: by way of direct proof, we may mention the experiments of Sir E. Home, in which stimuli applied to the sympathetic nerve increased the number of pulsations in the carotid artery.†

The general union and sympathy effected by this system among the most distant parts of the body, is likewise too familiar to our readers to require much comment. To give a few examples: Titillation of the nostrils excites sneezing, because the nasal nerves communicate with the sympathetic, through the medium of the vidian. Vivid light likewise sometimes excites sneezing, because the impressions perceived by the retina, and transmitted to the ciliary nerves, is continued to the sympathetic by the nasal branch of the fifth pair. The anastomosis between the fifth nerve and the sympathetic likewise explains the grinding of the teeth and itching of the nose from worms in the intestinal canal. A calculus in the kidney excites vomiting and other derangement of the digestive organs; while

---

* *Journal de Medecine*, 1816.
† *Philosophical Transactions*, 1814.

one in the bladder is comparatively harmless, as regards these functions: the explanation consists in the fact, that the nerves communicating between the kidneys and stomach are much more numerous than between the stomach and urinary bladder. The consent between the chylopoietic viscera and the retina is likewise unequivocally shown in many cases of indigestion. The great medium of sympathy, however, between the head and viscera of the thorax and abdomen is the par vagum, between which and the nerve under consideration numerous anastomoses take place in these cavities; one of the most important amongst which is the branch of the nervous vagus which joins the solar plexus, and is described by WRISBERG under the name of *fascia communicans.** Lobstein gives this an appellation somewhat more lengthy, and almost amounting to a description; he calls it *ramus magnus anastomoticus abdomino-cephalicus.* He states that the greater part of the gastro-pulmonary nerve reaches the semilunar ganglion in an uninterrupted course; and this ganglion again sends out branches, which are so united with those of the par vagum, that it is impossible to point out where the one begins and the other ends.

On the subject of the use and functions of the sympathetic nerve in the affections of the mind, we shall trespass but little on the patience of our readers. The only difference we can discover between the doctrines of Lobstein and Bichat is this: the latter considered the gastric viscera the seat of the mental impressions, so that, if the stomach was placed in the pelvis and the liver in the chest, their sympathies would be changed;—Lobstein, on the contrary, thinks the mental impressions operate through the medium of the semilunar ganglia, which he denominates "cerebrum abdominale;" and, consequently, that the mere locality of the viscera is of little moment, so long as they are supplied with nerves from this source. We rather prefer the opinion of Lobstein, but we do not think the discussion worth entering upon. Neither are we more disposed to trouble our readers with any inquiry into the *mechanism* by which the influence of the sympathetic nerve is carried on; the question evidently involves that of nervous energy in general, which it has been the fashion of late to identify with galvanic electricity. This is probably as good a tub to amuse the whale withal as any other; although we confess ourselves utterly at a loss to comprehend how the fact of electric stimulus, calling nervous power into action, identifies with the power so excited, any more than the application of other stimuli, such as volatile alkali or the prick of a needle. The only superiority possessed by electricity, is that it can be applied with more convenience

* *Comment. Gœtting.* vol. xv.

in a continued stream of stimulation, so giving the appearance of being itself that which it does but call into action.

Lobstein also dislikes the system which reduces the animal body to a galvanic pile, but has not the virtue to abstain from speculating with regard to this influence, which, if it be, as he supposes, a *gas*, seems at least to be one of a nature too subtile and too fine for the grasp of human intellect. "Istud principium (says our author,) sub specie halitus seu vaporis nervorum stamina pervadere suspicor ac proprium gaz constituere nervosum atque imponderabile, quod nulli assimilari potest fluido, neque *electrico, neque magnetico.*"

### *Section Third—Pathological.*

Our limits oblige us to be very brief on this branch of the subject. Among the diseases enumerated by Lobstein, as connected with morbid conditions of the sympathetic nerve, are hypochondriasis, hysteria, melancholia, mania, angina pectoris, asthma, syncope, incubus, latent gout, and perhaps intermittent fever. We next have the diseases sympathetically produced from primary disorder in the ganglionic system: these are, hemicrania and head-ache from indigestion, tinnitus aurium (from the anastomosis in the tympanum, described by Jacobson,) convulsions from worms, drowsiness bordering on apoplexy, from intestinal accumulation or obstruction, and various others, known and acknowledged by most practitioners of the present day. These we pass over, in order to leave room for the insertion of some dissections given by our author, and which we consider the most important part of his pathological discussion.

A female, of regular habits, had suffered from spasmodic and hypochondriacal symptoms from the age of puberty, and had twice been attacked with incomplete apoplexy, which left a slight paralysis of the right side of the face; at forty-two, she was married, and fell with child fifteen months after. At the eighth week of utero-gestation, a train of symptoms arose which subjected the patient to great misery. She had vomiting to such an extent that, for three months, she immediately rejected every thing taken into her stomach; no relief being afforded by any internal medicine or external application. During the last seven days of her life, even a drop of water, when swallowed, was refused by the stomach. The internal fauces and the mouth first became inflamed, and subsequently mortified, from the continued excitement of vomiting; and the patient's fingers, in consequence of her sometimes putting them into her mouth, were ulcerated by the acrid discharge. The most distressing symptom of all, however, was a burning pain along the course of the spine and in the lower part of the right hypochondrium, by which a constant jactitation was produced, and the strength of the patient exhausted. The only means by which this pain could be at all alleviated was dry friction, so that the skin was completely excoriated. This unfortunate patient at length died, in a state of miserable extenuation. The body was examined forty-eight hours after death. Much attention was given to the brain, but no unnatural appearances of any kind discovered. The head was thought to be rather under the usual size, but the patient had not been deficient in mental endowments. The thoracic viscera were in a state equally sound; the stomach presented no organic lesion, although there had been black vomiting; neither did the rest of the alimentary canal, nor the urinary system, differ from their healthy condition. The liver was soft and livid. The uterus, in the fifth month of pregnancy, showed no marks of disease, except some fibrous tumors in its substance, equaling walnuts in size. The cervix uteri was hard, perfectly closed at its external orifice, by which it had resisted the attempts made to produce abortion, and thus get rid of the vomiting by removing its cause. The fœtus was healthy, and in its natural situation. The viscera were now all removed, and the semilunar ganglia cut out: "it was not, indeed, converted into a foreign substance, but its colour was intensely red, which was regarded as true and genuine inflammation, by some men of experience and great cultivators of anatomy, to whom I communicated this observation. The inflammation was so obstinate, that the ganglia were but little paler after three days' infusion in cold water; and I took care to have the right ganglion drawn in this state, and represented in the seventh plate of this treatise. Its upper part is of a vivid red; but the inferior, from whence the mesenteric branches go off, is more livid. The splanchnic nerve appeared to me much broader than usual, before its entry into the ganglion." We do not think the plate very well executed, and are disposed to trust more to the description than the representation. The author conjectures that this inflammation had been long present, in a chronic form, and that it may have given rise to those nervous and hypochondriacal affections to which we have above alluded: the burning pain along the vertebral column is, of course, attributed to the state of the semilunar ganglion; but it is to be regretted that the spinal cord itself was not examined.

The body of a girl was examined, who had been attacked with epidemic cough, which was converted, by metastasis, first into spasmodic vomiting, and then into atonic convulsions, which could not be overcome by any remedial means. In this case the left portion of the solar plexus was inflamed; the right being healthy. In order to ascertain the fact in a more satisfactory manner, the part was steeped for some days in water; at the end of which time the redness had passed into a yellowish tinge, confined to the portion supposed to be inflamed. The cause of the convulsions was in vain sought for in the brain in this case, which the author explains by regarding them as sympathetic.

AUTENRIETH states, that he has found the par vagum inflamed in its whole course through the thorax, in the case of a girl cut off by hooping-cough: the sympathetic had likewise suffered some change.*

The following cases were communicated to M. Lobstein by Dr. ARONSSOHN:

A man, aged forty-seven, had a fibro-cartilaginous tumor, which adhered loosely to the spine, cut out. In two years he returned to the hospital, having another tumor on the same site: he was seized with tetanus, and died in two days. On opening the body, were found, first, a vascular sac well filled with blood, at the surface of the medulla spinalis, and a quantity of serum effused within the sac formed by the dura mater; secondly, very distinct inflammation of the semilunar ganglia.

A woman, aged thirty-six, was afflicted with vomiting during the whole period of gestation, in her second pregnancy. This continued after delivery, and at length became milder, from a furfuraceous eruption which occurred in the chest and arms. To this succeeded swelling and inflammation of the left knee, accompanied with diarrhœa; on the supervention of which the vomiting, which had lasted nearly three years, disappeared. The patient died exhausted by suffering and hectic fever. On opening the body, the villous coat of the stomach was found inflamed and thickened, particularly near the pylorus, and the semilunar ganglia affected with genuine phlogosis, *(ganglia semilunaria genuina phlogosi correpta.)*

In a boy, of ten years old, who died with symptoms of anxiety and oppression in the chest, and rattling at the pit of the stomach, in consequence of the retrocession of an exanthematous eruption, the author found a part of the trunk of the sympathetic nerve closely inflamed, with phlogosis of the ninth and tenth thoracic ganglia, and of the two anastomosing branches sent from the costal nerves. These marks of disease in this class of nerves are, as the author justly observes, too important to be despised; at the same time, we fear that much additional information must be collected on this subject before they can become available, either for diagnostic or curative purposes.

Pursuing his inquiries with regard to the nerves of the lungs, M. Lobstein informs us that he discovered another degenerative structure, apparently peculiar to them. In that species of pe-

---

* *Tubinger Blætter für Naturewissenschaft, &c.* 1815.

ripneumonia in which the lungs become red and slightly indurated, a state which he proposes to call *spleenification;* the nervous twigs accompanying the ramifications of the bronchiæ are likewise red, rather more tumid than usual, and so much softer that they were torn by the slightest force. This, however, seems to be the only diseased state of lung in which he has detected any organic lesion of the nerves; none having been observable in ulcers or vomicæ; neither in the hepatised lung nor the tuberculated, of whatever kind this might be. One case in particular is mentioned, in which the body of a woman was examined, whose lungs were crowded with tubercles, the largest equalling an orange in size, the smallest the kernel of a cherry; the whole weighing nine pounds, without the heart. The tubercles were all formed of a matter resembling the brain of the fœtus, not included in any sac, but in contact with the surrounding paranchymatous structure, which was perfectly healthy. The upper lobe of the right lung was in a manner converted into a large sac, containing this pultaceous matter, mixed with coagulated blood. It is worthy of remark, that the pulmonary nerves, examined with the greatest care, were found to differ in no respect from their healthy condition: they entered the diseased parts, and traversed them, without undergoing any alteration.

The author goes on to state that he has frequently seen the branches composing the anterior and posterior pulmonary plexuses, compressed, displaced, and drawn aside by lymphatic glands in a state of disease, and yet more frequently by calcareous tubercles closely united to the bronchia. When the glands above mentioned abounded with black matter, the nerves were sometimes tinged of the same colour, and somewhat softened. With respect to the stony concretions, he has found many adhering so firmly to the nerves, that they could not be separated from them without considerable force; and this he has likewise remarked in the solar plexus, at the part next the suprarenal ganglion. On one occasion he found a concretion as large as a cherry-stone imbedded in the trunk of the nervous vagus, and separating its fibres from each other; the functions of the lungs and stomach having remained unimpaired.

In the cardiac nerves, his observations have principally related to the aneurism of the aorta; these being but seldom altered by diseases of the heart. In dilatation of the aorta, he has found twigs of nerves received, as it were, into grooves in the thickened coats of the vessel; whereas, in the natural state, they are only agglutinated to the parts. They have appeared elongated, (as, indeed, might be expected from the augmented volume of the artery,) and redder than natural: in those cases distracting burning pain had been present, which could not be accounted for otherwise than from their displacement and inflammation.

In the case of a woman who laboured under aneurism of the aorta, with hydrothorax and hydrops pericardii, the phrenic nerve and par vagum were found contained and compressed in a dense false membrane, thrown out by the aneurismal sac; while the principal cardiac nerves were altogether wanting. Lobstein argues that the compressed state of the former, interrupting the flow of nervous power, and the entire absence of the latter, may have had a considerable share in producing the other morbid phenomena. With regard to the compression of the phrenic and gastro-pulmonary nerves, it is to be remembered that the disease, as described before us, must have previously attained a great extent before such compression could be effected.

The inflammation peculiar to the nerves, and that which spreads to them from their vicinity to other parts, are to be distinguished: in the latter case, the inflammation is not so intense, and disappears on infusion in water. In violent pleuritis, Lobstein has found the thoracic ganglion not increased in bulk, nor at all thickened, yet having its surface covered with a beautiful vascular web: the redness, however, did not penetrate into the interior; for the ganglion, being cut through, exhibited the same healthy appearance as the others. It seems, however, from a case which follows, that part of the jejunum, being included in a strangulated hernial sac, had become deeply inflamed; and the nerves of the part were also redder and more pulpy than natural. Indeed, the branches of the sympathetic appear to become thickened in various diseases: our author has seen the nerves constituting the suprarenal capsule much enlarged in a disease affecting the kidneys; and Dr. Duncan, of Edinburgh, has given another, in which the sympathetic had attained three or four times its usual size in a case of diabetes, in which the urinary bladder was much dilated,* from its entrance into the abdomen until its termination in the pelvis.— Besides increasing in bulk, our author is of opinion that the nerves actually increase in number. 1. He has detected many twigs of nerves in the spermatic cord, evidently belonging to the blood-vessels, and one going to the vas deferens. In this case he assured himself, by microscopic examination, that he did not mistake lymphatics for nerves. 2. In an organic disease, in which the epididymis was converted into a cyst, containing serous fluid, he found abundance of nerves going to the testicle. 3. In a thyroid gland, which was much enlarged, and weighed

---

* *Reports of the Practice in the Clinical Wards of the Royal Infirmary of Edinburgh.* 1818.

four pounds, he found an immense number of nerves, *(perquam numerosi.)* The nervous trunks arising from the laryngeal nerve and the olivary ganglion entered each side of the gland, accompanying the superior thyroid arteries. Each arterial branch had a companion from the nerves, not united with its coats, otherwise than through the medium of cellular substance. All the ramifications of the nerves communicated with each other, forming a beautiful plexus; they united with those of the opposite side, constituting a kind of crown at the upper margin of the gland. From thence branches descended over the external surface, one of which anastomosed with an ascending twig of the recurrent nerve. Nothing unusual was remarked in the structure or appearance of any of these ramifications. But, if the number of nerves supplying any given part may thus be occasionally increased in number, so in other instances they seem to be diminished. This takes place when the organ which they supply becomes destroyed, so as not to require so much nervous energy.

In a case of colica pictonum, and another of schirrus of the stomach, the author found small yellowish purple tubercles in the solar plexus. Tumors frequently form in various parts of the abdomen, and, in some such instances, Lobstein informs us that he has found the nerves torn across by the distention. In an instance of this kind, where the tumor was situated behind the hypogastric plexus, this was divided into two portions, or superior and inferior: the former ended at the tumor, presenting a gangliform appearance, like that exhibited by the nerves of a stump after amputation; it was covered with a cicatrix. The other extremity, though separated by a finger's breadth, was not wasted. In a man, aged forty-seven, who had been seized with sudden cardialgia, and dreadful pain of the back extending between the shoulders, followed by obstinate constipation and excruciating tormina, under which he sunk at the end of seven weeks, both nervi vagi were found lacerated by a tumor in the epigastric region, which adhered firmly to the lesser curvature of the stomach.

The last diseased appearance of this nerve, to which we have to call the attention of our readers, occurs in the fœtus, and has been discovered by Lobstein in three embryos at the fourth month. It consists in tumefaction and yellowness of the sympathetic in the thorax. This colour, which is a golden yellow, purvades the internal structure of the nerve, and is neither removed by water nor alcohol; but is destroyed by exposure to the sun's rays. The medulla spinalis presented the same degeneration, its whole substance being yellow, and appearing, on microscopic examination, as if sprinkled with a very fine shining powder. To this disease, the nature of which is quite unknown,

Lobstein proposes the name of *Kirronoson,* (from κιῤῥος, gold-coloured, and νοσος, a disease;) and sums up his dissertation in the following conclusions:

"From all we have said concerning the nerve, the structure and uses of which we undertook to illustrate, it follows,—first, that it forms a peculiar system in the animal economy, and is not produced from the brain nor spinal marrow, although it freely communicates with them; secondly, it is designed for certain definite functions; thirdly, not only is it capable of being affected in its powers and actions, so as to produce numerous diseases, but likewise of exhibiting in its structure changes which are perceptible to the senses."*

---

ART. V.—*An Essay concerning Tussis Convulsiva, or Whooping-Cough; with Observations on the Diseases of Children.* By BENJAMIN WATERHOUSE, M.D. Member of many Medical, Philosophical, and Literary Societies in Europe and America; and late Professor of the Theory and Practice of Physic in the University of Cambridge.—pp. 152. Boston (America), 1822.

OUR Transatlantic brethren have taken mortal offence at an expression which once fell from the Edinburgh reviewers,—"Who ever goes to an American play, or who ever reads an American novel?"—"What does the world yet owe to American physicians and surgeons?" The expression was a harsh one, savouring too much of nationality, and might certainly have been spared; but, having been used, it cannot be denied to have had some real foundation. We cannot, at this moment, call to mind any one leading principle, in physiology or pathology, any one acknowledged improvement in surgery, or any one remedy of general efficacy, proposed by an American practitioner. Why this should be, it is not our purpose to inquire. We have only stated it, because it was the impression upon our minds when we sat down to the perusal of the work before us. How far this volume has succeeded in removing that impression from our minds, and to what extent it is likely to add to the fame of the author, and the renown of American pathologists, the sequel will show. We know nothing more of Dr. WATERHOUSE than what the title-page teaches us: he is evidently a man who has lived long, and seen a great deal of practice; and, having been professor of the theory and practice of physic in the University of Cambridge, he is entitled to some degree of consideration. We shall now, therefore, attempt to give our readers a notion of the peculiar views which this gentleman entertains, and has long been in the habit of teaching, concerning

---

* See an interesting paper on the *Ganglionic System*, by Dr. COPLAND, in the London Medical Repository.

the nature and cure of *whooping*-cough. In compliment to the author, we shall spell the word with a *w*, though we cannot see why, upon the same principle, we ought not to write *whowl* and *whollow* for *howl* and *hollow*.

The author commences with remarking that whooping-cough, though a very frequent disorder, is very little understood; and he adds, that it would be difficult to direct the young practitioner to any writer for such information as might be sufficient to satisfy himself, and benefit his patients. He further states (page xiv.) that, from the recorded opinions of the best modern practitioners, we cannot learn any thing like a steady principle, or a fixed and consistent practice. It is very true that Dr. Waterhouse does not undertake to clear up all these difficulties: he means only to *agitate the matter afresh*, to excite inquiry, and to *aid the growth of knowledge*. With these judicious ends in view, he enters upon his investigation.

His description of the initiatory symptoms of whooping-cough, as they occur in the adult subject, appears to us ably drawn up, and worthy the attention of the reader.

"To a bystander the disorder appears to come on with the ordinary symptoms of catarrh; but, to the attentive adult patient, its approach is intimated by an universal soreness of the flesh, especially of the abdomen, not unlike the symptoms of an incipient peritonitis. This tenderness extends from the insertion of the diaphragm, in the xiphoid cartilage of the sternum, to the pubes and groins; and is accompanied with a sense of weariness, especially in the loins, together with an uneasiness, stricture, or sense of twisting in, we presume, the upper orifice of the stomach. In some, if not in all, there is a sense of heat in the direction of the ureters, and somewhere about the bladder.

"During the soreness of the flesh, which lasts three or four days, there is a short, hacking, husky effort, hardly amounting to a cough. It seems a peculiar affection of the œsophagus, or pharynx; and this cough to be an instinctive effort to rid, or diminish, this slight but disagreeable feeling. As yet, there is no soreness or painful affection of the chest, nor sense of stuffing as in catarrh, nor remarkable difficulty of breathing, neither any sensation of membranous tightness during the action of coughing, nor tender spot in any part above the diaphragm. The tenderest place is the rim of the belly, and in the groins. In an advanced state of the disorder, the pain and distention is so great in the groin, as to excite in some apprehensions of rupture, during the violent convulsive paroxysms.

"On the appearance of symptoms resembling catarrh, the soreness of the abdomen gradually lessens, and the characteristic symptom of this distemper begins moderately to manifest itself. I mean the convulsive hiccupish cough. During the symptoms of this early period, there is more or less fever, with occasional head-ache, especially on sudden exertion, or after eating freely. The patient has a greater disposition to sleep in the day-time than usual, while the habitual sleep of the night is disturbed with unpleasant dreams. He feels, at times, more than usual coldness in his back and extremities. Although his appetite is now and then keen, it fluctuates, and his general condition denotes a febrile state: his urine is high-coloured, and small in quantity; his bowels, for the most part, bound, but sometimes otherwise; while the skin has that hot and dry feel observable, very often, on the first approach of dysentery.

"Somewhere between the fourteenth and twentieth days, the distemper assumes its peculiar symptoms, which is a singular retch, catch, hiccup, or cough, accompanied with a sort of half-crowing sound, denominated 'a whoop;' of which no author yet consulted has given me a satisfactory explanation. We now consider the disorder to be formed, and the convulsions to have taken their stand, in order to recur three, four, or five times in the course of twenty-four hours. Of these fits, or paroxysms, the one that occurs about two o'clock at night, which is usually after the first nap, is the most violent. The fits may recur at any time, on sudden surprise; suddenly excited passions of the mind; eating any dry morsel, as biscuit; attempting to drink a glass of madeira, or any other very strong wine; or running, or walking too fast." (p. 22, 23.)

The further description of the symptoms of the disease is good, and may be consulted with advantage; but we hasten to the second and third chapters, entitled "of the Seat of the Whooping-cough, and of the Mucous Membrane, with a disquisition on the Diaphragm," that the reader may come at once upon the leading feature of this work, and be enabled to form for himself some estimate of the merits of our author as a pathologist.

His first attempt is to prove a *distinction* between whooping-cough and those affections in which cough is a prominent symptom, viz. catarrh, influenza, croup, and bronchitis. He states that,

"In whooping-cough, the subject can fill his lungs with air, by a deep inspiration, and long retain it there. He can use the blow-pipe, play on wind instruments of music, without creating any remarkable difficulty of breathing, exciting any pain, or occasioning a fit of coughing. Moreover, patients in the height of whooping-cough have rode in a thick atmosphere of driving dust, without bringing on the convulsive cough; while the same persons, on the same day, swallowing certain warm liquors, tea, coffee, or wine, were instantaneously thrown into a convulsive paroxysm. Any drink, even warm milk, is apt to produce the fit, unless swallowed with premediated caution." (p. 43.)

On the other hand,

"When a patient in bronchitis attempts a deep inspiration, sufficient to extend the chest to its fullest capacity, the effort is interrupted by a common cough, unaccompanied by any thing like a whoop. Beside, in lying down, the difficulty of breathing in the bronchitic patient is much greater. Moreover, there is a thick, whitish, frothy mucus in the one, that is not in the other; with violent pain across the eyebrows, from temple to temple; loathing of food, great thirst; hard, full, and crowded pulse; and a high fever, which belong not to whooping-cough. The deep anxiety, distressful countenance, inability of breathing but in an erect posture, and the little relief by expectoration, all lay so strong a line of demarcation between bronchitis and whooping-cough, that we are surprised at the analogy, or rather identity, contended for by some late writers in England and in Germany." (p. 45.)

The author's notion is that the lungs and air passages, the *systema spiritale pneumonicum*, as he styles it, is not primarily, but secondarily and sympathetically affected in whooping-cough, and that the true seat of the disease is the œsophagus, stomach, duodenum, and diaphragm. So impressed is he with this belief, that he actually declares that this congeries of organs is "*the original and primary seat of nearly all the internal distempers that flesh is heir to.*" Of this præcordial region, Dr. Waterhouse has formed the most extravagant ideas. After stating what no one certainly can deny, that with a healthy state of the digestive functions we enjoy strength, activity, and cheerfulness, and that with languishing digestive functions the contrary of all this happens, he proceeds:

"But this is not all. 'Tis but a partial view of that central influence, emanating from the stomachic and phrenic region; for, while the stomach is digesting solid food, the lungs is digesting air. When we inhale the atmospheric air, the respirating organs, in the action of breathing, separate that portion of the inspired mass, which, under the name of spiritus, vital air, or oxygen, enters the blood, vivifying and animating the whole system, corporal and corporeal." (p. 47.)

What the author means by this latter phrase, or whether it is an error of the press, we have no means of judging. He is so carried away by his admiration of the great *centrum ovale* of the præcordia, the diaphragm, that words fail him in his attempt to explain its importance in the animal economy.

"The *tendon*, so called, *for it seems to merit a nobler name,* in the centre of the diaphragm, is determined in its shape by the extent of these fleshy bellies; for the great muscle above almost surrounds the central tendon; the smaller muscle below meeting it, the two divisions give it a pointed form behind. The middle line of the tendinous centre is fixed by the membrane which divides the thorax into two; the two sides go upwards into the two sides of the chest, each with a form resembling the bottom of an inverted basin; their convexity reaching within the thorax, quite up to the level of the fourth true rib.

"The proper centre of the diaphragm is fixed by this connexion with the mediastinum, that its motion might not disorder the action of the heart, which rests upon this point, and whose pericardium is fixed to the tendon: but the convexity of either side descends and ascends alternately, as the diaphragm contracts or is relaxed; so that it is chiefly these convexities, on either side, which are moved on breathing. And thus is the diaphragm composed of one great and circular muscle before, of one smaller circular muscle behind, and of the triangular tendon betwixt them; while both, in its fleshy and tendinous parts, is perforated by several vessels, passing up and down reciprocally between the thorax and the abdomen. *Is there any thing under the name of muscle, in the human body, like unto this?*" (p. 51.)

"Such is the diaphragm, as far as the eye can see. Its anatomy, if duly considered, must enlarge our ideas of its importance to the body; yet there are several things in our constitution hidden from the eye that are evident to reason, and in no part more than this. That the ancients had an exalted idea of this organ, is clear from their giving it a name synonymous with *mind.* It appears too complicated in structure, curious and singular in its attachments and manifold connexions, to be regarded merely a muscle. The lungs belong to it; the heart rests upon it, as upon a floor. It embraces that remarkably sensible spot, the cardia, or upper orifice of the stomach, forming there a species of sphincter. It is connected to the liver by a hem, made by the juncture of the pleura and peritoneum; to which we may add its firm attachment to that enigmatical organ, the spleen, with its placenta-like structure. When to these particulars we subjoin the course of the sympathetic and phrenic nerves, with the ganglia thereon depending, and the well-known fact that phrensy is the consequence of inflammation of this organ, we must believe that the diaphragm has a more powerful and extensive agency in the economy of man than is generally allowed, or perhaps suspected.

"We cannot every where see the connexion between structure and function. We can see how it is that the jaws bite, the teeth masticate, and the œsophagus swallow; but how the stomach, a mere bag, can convert a vegetable into our own animal nature and substance, we know not; and so of the diaphragm, with its matchless structure and manifold connexions." (p. 52, 53.)

"Have we not reason for believing that the centre of the diaphragm, probably where the aorta and vena cava pass from above downwards, and from beneath upwards, is the seat, source, or receptacle of that nervous energy which is suitable to the organic life and action of all the abdominal apparatus? The idea of the throat, stomach, and intestines being *one great gland*, receives rather support than injury from this opinion. John Hunter used to tell us that a very severe blow on the pit of the stomach killed the *whole man* so instantaneously and outright, that the muscles had not time to become rigid.

"Have we not, then, reason for concluding that this diaphragmatic region is the citadel, or focus, of that nervous power to which the whole alimentary canal and assistant viscera, together with the urinary and uterine organs, depend for support?

"This region, or colony of the brain, maintains a remarkable connexion with the mind. It is alternately the seat of hilarity and woe. Here it is that moping melancholy intrenches itself deep against every thing joyous. When dejection sits too heavy on the diaphragm, it endeavours instinctively to relieve itself from the incumbent load, by a heaving sigh. Should anxiety increase to affliction, the sigh shakes the vocal organs with a groan. If affliction progress to agony, then short and convulsive sobs follow; and, when distress is augmented to anguish, a violent scream is the last effort for mitigating the feeling of wretchedness; after which follow fainting and death. It was, doubtless, from observations of this sort that led physicians and anatomists to call that great nerve, which, entering the diaphragm, strikes out like radii from its centre, the *phrenic nerve*." (p. 54.)

These pretty copious extracts will serve to give our readers a good notion of the style of the author, as well as of his peculiar views in pathology.

[To be continued.]

